# The effect of manganese nanoparticles on apoptosis and on redox and immune status in the tissues of young turkeys

**DOI:** 10.1371/journal.pone.0201487

**Published:** 2018-07-31

**Authors:** Jan Jankowski, Katarzyna Ognik, Anna Stępniowska, Zenon Zduńczyk, Krzysztof Kozłowski

**Affiliations:** 1 Department of Poultry Science, Faculty of Animal Bioengineering, University of Warmia and Mazury, Olsztyn, Poland; 2 Department of Biochemistry and Toxicology, Faculty of Biology, Animal Sciences and Bioeconomy, University of Life Sciences in Lublin, Lublin, Poland; 3 Institute of Animal Reproduction and Food Research of the Polish Academy of Sciences, Olsztyn, Poland; University of Illinois, UNITED STATES

## Abstract

The aim of the study was to determine whether the use of Mn nanoparticles would make it possible to reduce the level of this micronutrient added to turkey diets without adversely affecting growth performance, antioxidant and immune status, or apoptosis. The experiment was conducted on 6 groups of turkeys with 10 replications in a two-factor design with 3 dosages of manganese, 100, 50 and 10 mg/kg, and 2 sources, manganese oxide (MnO) and manganese nanoparticles (NP-Mn_2_O_3_). The study showed that irrespective of the form of Mn used, reducing the Mn level recommended by British United Turkeys for supplementation of the diet of young turkeys from 100 mg/kg to 10 mg/kg increases the content of this element in the blood with no adverse effect on growth performance or the immune system. The reduction in Mn supplementation in the form of NP-Mn_2_O_3_ from 100 to 50 and even to 10 mg/kg of turkey diet has no negative effect on antioxidant defence in young turkeys. A 50% reduction of the recommended Mn level in the form of MnO enhances lipid oxidation processes. Replacing MnO with NP-Mn_2_O_3_ in the turkey diet probably can increase apoptosis in young turkeys. On the other hand, irrespective of the form of Mn used, reducing supplementation of the turkey diet with this element from 100 to 50 and even to 10 mg/kg probably can reduce apoptosis.

## Introduction

Manganese (Mn) is one of the micronutrients necessary for the proper growth, development and functioning of living organisms. It is a cofactor and activator of many enzymes, such as galactosyltransferase, agmatinase, arginase, glutamine synthetase, pyruvate carboxylase and superoxide dismutase, which are responsible for proper bone development, cell structure, metabolism, the mitochondrial antioxidant system and cell death [1,2].

Due to these physiological functions of Mn, poultry diets are enriched with this element, usually by adding various chemical forms of Mn, including MnO and Mn_2_O_3_ [3]. It is accepted that in the case of growing turkeys, the addition of Mn to the diet should amount to 60 mg kg^-1^ of feed throughout the rearing period [4]. British United Turkeys (BUT) recommendations are much higher, amounting to 120 mg kg^-1^ in the first 4 weeks of rearing and 100 mg kg^-1^ after 4 weeks. The risk of negative consequences of both excessive and insufficient Mn in poultry diets is relatively high. Excess Mn accumulates mainly in bone tissue, but also in other organs, such as the liver, kidneys and brain [5–7]. Ionic Mn also interferes with chemical synapse functions and is able to cross the blood—brain barrier, and therefore chronic MN exposure may eventually lead to neurotoxicity and Parkinson-like symptoms [8–12]. At the cellular level, Mn preferentially accumulates in the mitochondria, where it disturbs oxidative phosphorylation and increases the production of reactive oxygen species (ROS) [13]. Excessive ROS production induces oxidation of membrane polyunsaturated fatty acids, generating a number of lipid peroxidation products and inducing oxidation of proteins and DNA [14]. However, it is not entirely clear how excess Mn inducing ROS formation affects the lymphatic system and the immune response in birds [15]. According to Liu et al. [16], one possible reason for the reduction in Cu content in the tibia and liver and in immunity is that excessive Mn in the diet causes a disturbance in the proportions of trace elements in lymphoid organs of broiler chickens. A study by Collins and Moran [17] also confirms that a high level of Mn supplementing the diet affects the bioavailability of other minerals, masking the benefits of their supplementation. Excess Mn may disturb the balance of trace elements in the immune organs. This imbalance can induce immune suppression and oxidative stress responses. In addition, it can increase apoptosis in poultry [16, 18–20].

On the other hand, there are reports indicating that Mn deficiency in poultry diets may disturb normal growth and reproduction [21]. Research conducted by Qin et al. [22] indicates that Mn is a trace element necessary for the normal growth and development of bird embryos. Because Mn regulates the metabolism of hormones involved in bone metabolism [23, 24], a diet low in Mn may increase the incidence of perosis in poultry and decrease the length of the tibia [25, 26]. It has also been observed that a diet low in Mn may increase the production of thin-shelled eggs in laying hens [27, 28].

The practice of adding inorganic forms of Mn to poultry diets in amounts usually exceeding NRC recommendations results in the excretion of large amounts of this element into the environment, causing contamination [29–31]. One strategy to reduce Mn secretion into the environment, described by Ghosh et al. [32], is the addition of a suitably large number of microbial phytase units (500 FTU/kg) to the diet. Phytase hydrolyses phytic acid to inositol and improves absorption of Mn [33]. In the last decade, researchers have been increasingly searching for alternative chemical forms of Mn, mainly organic compounds of this element [3], which are highly bioavailable and which reduce the amount of Mn released into the environment. It is also believed that the addition of smaller amounts of Mn in the form of nanoparticles to turkey diets may be a way to improve the absorption of Mn from the diet and reduce its excretion into the environment. Nanoparticles, unlike traditional macro- or microstructures, display much greater physical activity and chemical neutrality. Due to their small size, they have the ability to easily penetrate biological membranes, and their biological activity results from a large surface area enabling direct contact between the molecules and target cells [34]. It is unknown, however, whether Mn nanoparticles (potentially better utilized than the commonly used inorganic forms of Mn) can be added to turkey diets, in the amount recommended by BUT or in even smaller dosages, without negatively affecting production results or the immune and antioxidant status of young turkeys.

Therefore, the aim of the study was to determine whether the use of Mn nanoparticles would make it possible to reduce the level of this micronutrient added to turkey diets without adversely affecting growth performance, antioxidant and immune status, or apoptosis.

## Material and methods

### Animals

The experimental procedure was approved by the Local Ethics Committee for Experiments with Animals in Olsztyn, Poland (approval no. 30/2015). The subject of the study was 1080 day-old Hybrid Converter turkey hens purchased at the Grelavi hatchery in Kętrzyn Poland. The birds were randomly assigned to 60 pens with an area of 4.0 m^2^, with 18 individuals per pen. The pens were lined with wood chips. The stocking density of the birds was 4.50 birds/m^2^. The birds had unlimited access to feed and water, and the environmental conditions were adjusted to the age of the birds and Hybrid recommendations. The experiment was conducted on 6 groups with 10 replications in a two-factor design with 3 dosages of manganese, 100, 50 and 10 mg/kg, and 2 sources, manganese oxide (MnO) and manganese nanoparticles (NP-Mn_2_O_3_). The difference between the total Mn content in diets and the amount of supplement used (100, 50 and 10 mg/kg) shows that the basic diet components contained a total of about 45 mg Mn/kg of feed. Manganese nanopowder (NP-Mn_2_O_3_, purity 98+%, 40–60 nm, spherical specific surface area 13.5 m^2^/g, bulk density 1.2 g/cm^3^) was purchased from SkySpring Nanomaterials (USA). The feed was produced by Agrocentrum sp. z o.o. in two stages: (1) as basal feeds without the addition of a vitamin- mineral premix, and then (2) with vitamin mineral premixes containing the appropriate amount and form of Mn added to the feed for each experimental group (Table 1).

**Table 1:** Composition and nutritional value of compound feed fed to turkeys from 0 to 42 d.

**Components, g/kg**	
Wheat	521.1
Soybean meal	409.5
Soybean oil	19.0
Sodium sulphate	1.5
NaCl	2.0
Limestone	16.6
Monocalcium phosphate	16.4
DL-Methionine 99	2.9
L-Lysine HCl 78	4.7
L-Threonine 99	1.0
Feed enzymes^1^	0.3
Mineral-vitamin premix^2^	5.0
Calculated nutrient density, g/kg	
Crude protein	265.0
Crude fibre	29.0
Arginine	16.9
Lysine	17.0
Methionine	6.5
Methionine+Cysteine	11.0
Threonine	10.3
Tryptophan	3.2
Ca	11.5
Pav.	5.5
Na	1.5
AME, kcal/kg	2750
**Amount of Mn added to feed**	**Analyzed content of Mn, mg/kg**
100 mg/kg MnO	140
100 mg/kg NP-Mn_2_O_3_	139
50 mg/kg MnO	96
50 mg/kg NP-Mn_2_O_3_	93
10 mg/kg MnO	75
10 mg/kg NP-Mn_2_O_3_	70

^1^ Phytase—750 FTU/kg, xylanase—300 FXU /kg

^2^ Mineral-vitamin premix per kilogram of diet (without Mn supplement): vitamin A—15,000 IU; vitamin D_3_−5000 IU; vitamin E—100 IU; vitamin K_3_−4 mg; vitamin B_1_−5 mg; vitamin B_2_−15 mg; vitamin B_6_−6 mg; vitamin B_12_−0.04 mg; nicotinic acid—100 mg; calcium pantothenicum—32.7 mg; folic acid—4 mg; choline—700 mg; biotin.—0.35 mg; Se—0.3 mg; Fe—60 mg; Zn—100 mg; Cu—20 mg; J—1.5 mg; Ca—1.04 g

AME-Apparent Metabolizable Energy

### Growth trial and sample collection

Feed intake, body weight gain, and the feed conversion ratio were determined during 6 wk. A pen of 18 birds was considered as an experimental unit for the purposes of determining growth performance parameters. At the end of the 42th day of life, 10 birds representing the average body weight of each group were selected, tagged, and fasted for 8 h. Blood samples were taken from 10 birds from each group (1 bird for each replication) with body weight similar to the treatment average. Birds was slaughtered in a processing plant 8 h after feed withdrawal (the Faculty of Animal Bioengineering’s slaughterhouse, the University of Warmia and Mazury in Olsztyn). The equipment of the slaughterhouse and all applied procedures gained approval of the Local Animal Care and Use Committee (Olsztyn, Poland; permission number 30/2015). The birds were electrically stunned (400 mA, 350 Hz), hung on a shackle line and exsanguinated by a unilateral neck cut severing the right carotid artery and jugular vein. After slaughter, the carcasses were scalded, plucked, and eviscerated. The redox status of the liver determined for 10 samples collected from each group.

### Laboratory analysis

The content of total cholesterol (TC), triacylglycerols (TG), urea (UREA), uric acid (UA), total protein (TP), albumin (ALB), glucose (GLU), creatinine (CREAT), calcium (Ca), phosphorus (P) and the activity of alanine aminotransferase (ALT), alkaline phosphatase (ALP), gamma-glutamyl transferase (GGT) were measured in blood plasma using an automatic biochemical analyzer (Plasma Diagnostic Instruments Horiba, Kyoto, Japan).

Markers of oxidative stress determined in the blood included lipid oxidation indicators, i.e. the concentration of lipid hydroperoxides (LOOH) and malondialdehyde (MDA), using kits produced by Cell Biolabs, Inc. (San Diego, USA). Activity of the enzymes superoxide dismutase (Mn-SOD) and glutathione peroxidase (GPx) in the blood of the turkeys was determined by spectrometry using Ransel and Ransod diagnostic kits manufactured by Randox (Poland). A diagnostic kit manufactured by Oxis International, Inc. (Portland, USA) was used to determine catalase activity (CAT). Activity of ceruloplasmin (Cp) in the blood plasma was determined using a Ceruloplasmin ELISA kit (Biomatik, Delaware, USA). Also determined in the blood were total glutathione (GSH+GSSG), using a Total Glutathione Assay (Cell Biolabs, Inc., San Diego, USA), total antioxidant status (TAS) using a diagnostic kit by Randox (Poland) and Caspase 3 (MBS261903), Caspase 8 (MBS094470), vitamin C content using an ELISA kit (Cell Biolabs, Inc. San Diego, USA). Immunoglobulins IgA, IgM and IgY and interleukin (IL)-6 in the blood were determined in an ELISA reader using assays from Elabscience Biotechnology Co., Ltd. (Houston, Texas, USA). As described previously [35], the following indicators of redox status were determined in the liver: the concentrations of lipid hydroperoxides (LOOH) and malondialdehyde (MDA), total glutathione (GSH+GSSG), activity of superoxide dismutase (Mn-SOD) and catalase (CAT) and content vitamin C in blood and liver. Manganese content in the samples of plasma and feed mixture was determined by inductively coupled plasma optical emission spectrometry.

### Statistical analysis

For the statistical analysis of performance parameters, a single pen (n = 10) was considered a replicate experimental unit. For the analysis of blood and liver parameters and growth performance, individual birds were treated as experimental units. The analysis of parameters was performed on 60 birds representing 10 replications from each of 6 experimental treatments. Two-way ANOVA was performed to determine the effects of Mn dosage (10, 50 and 100 mg/kg diet) and form (MnO or NP-Mn_2_O_3_) and the interaction between the two factors (dosage x source). The significance of differences between means in groups was estimated by Duncan’s multiple range test. Data were processed in the STATISTICA PL 12.0 application.

## Results

### Effect of manganese source

The results of two-way ANOVA showed that replacing MnO with NP-Mn_2_O_3_ decreased feed conversion per kg of turkey body weight (Table 2). Replacing MnO with NP-Mn_2_O_3_ caused an increase in GGT activity (*P* = 0.013) (Table 3), a decrease in CAT activity (*P* = 0.025) (Table 4) and the Casp 8 level (*P* = 0.001), and an increase in the Casp 3 level (*P*<0.001) (Table 5).

**Table 2:** Growing performance of turkeys in period of growth 1–42 days receiving different sources of manganese.

Treatment^1^	Body weight gain, g	Feed intake, g/bird/day	Feed conversion ratio, g/g
10 MnO	2358	96.7	1.658
50 MnO	2327	94.3	1.646
100 MnO	2366	95.7	1.643
10 NP-Mn_2_O_3_	2407	97.3	1.624
50 NP-Mn_2_O_3_	2310	94.8	1.648
100 NP-Mn_2_O_3_	2357	94.9	1.621
SEM*	23.32	1.021	0.043
Dosage			
10	2383	95.5	1.652
50	2319	96.5	1.634
100	2362	94.9	1.634
SEM	16.49	0.722	0.030
Source			
MnO	2350	95.8	1.650^a^
NP-Mn_2_O_3_	2358	95.5	1.630^b^
SEM	13.46	0.588	0.025
P-value			
Dosage (D)	0.059	0.280	0.239
Source (S)	0.329	0.765	0.048
Interaction D x S	0.181	0.148	0.809

In treatments 10 MnO, 10 NP-Mn_2_O_3_ and 100 NP-Mn_2_O_3_ 1 bird per group died.

^1^Turkeys in treatment 10 MnO received 10 mg/kg MnO, 50 MnO received 50 mg/kg MnO, 100 MnO received 100 mg/kg MnO, 10 NP-Mn_2_O_3_ received 10 mg/kg Mn_2_O_3_ nanoparticles, 50 NP-Mn_2_O_3_ received 50 mg/kg Mn_2_O_3_ nanoparticles, 100 NP-Mn_2_O_3_ received 100 mg/kg Mn_2_O_3_ nanoparticles.

^a-b^ means within the same column differ significantly (P≤0.05)

* SEM—for interaction D x S

**Table 3:** Indicators of biochemical indices in the blood of turkeys receiving different sources of manganese.

Treatment^1^	ALT U/L	ALP U/L	GGT U/L	UA μmol/L	UREA mmol/L	TC mmol/L	TG mmol/L	GLU mmol/L	TP g/L	Ca mmol/L	P mmol/L	Mn mg/L	CREAT μmol/L
10 MnO	6.26	1178	1.35	770	0.719	3.03	0.606	16.57	35.25	2.58	1.76	0.233	2.93
50 MnO	5.21	1295	2.69	396	0.876	3.36	0.621	17.16	37.10	2.80	2.07	0.173	4.28
100 MnO	5.29	1747	2.49	486	0.941	2.89	0.478	16.60	35.94	2.39	2.65	0.126	5.64
10 NP-Mn_2_O_3_	7.13	1176	4.91	400	0.578	3.16	0.595	17.48	37.59	2.54	2.04	0.310	4.41
50 NP-Mn_2_O_3_	6.30	1112	2.79	357	0.788	3.10	0.515	16.84	35.74	2.60	1.77	0.232	2.28
100 NP-Mn_2_O_3_	5.80	1810	3.78	373	0.778	3.32	0.561	16.76	36.73	2.67	2.77	0.111	6.36
SEM*	0.237	36.84	0.695	40.39	0.122	0.151	0.065	0.348	0.753	0.116	0.095	0.017	0.888
Dosage													
10	6.69	1177^b^	3.13	585	0.648	3.09	0.601	17.03	36.42	2.56	1.90^b^	0.271^a^	3.67^b^
50	5.76	1203^b^	2.74	376	0.832	3.23	0.568	17.00	36.42	2.70	1.92^b^	0.202^b^	3.28^b^
100	5.54	1779^a^	3.13	430	0.859	3.11	0.519	16.68	36.33	2.53	2.71^a^	0.119^c^	6.00^a^
SEM	0.168	26.05	0.492	28.56	0.086	0.107	0.046	0.246	0.532	0.082	0.067	0.012	0.628
Source													
MnO	5.59	1407	2.18^b^	551	0.845	3.09	0.568	16.78	36.10	2.59	2.16	0.187	4.28
NP-Mn_2_O_3_	6.41	1366	3.83^a^	377	0.714	3.19	0.557	17.03	36.68	2.60	2.19	0.239	4.35
SEM	0.137	21.27	0.401	23.32	0.071	0.087	0.037	0.201	0.435	0.067	0.055	0.010	0.513
*P*-value													
Dosage (D)	0.276	<0.001	0.843	0.371	0.255	0.684	0.533	0.618	0.993	0.390	<0.001	<0.001	0.018
Source (S)	0.188	0.671	0.013	0.169	0.246	0.473	0.850	0.437	0.397	0.888	0.721	0.083	0.931
Interaction D x S	0.928	0.552	0.089	0.527	0.961	0.136	0.430	0.285	0.100	0.175	0.075	0.292	0.194

^a-c^ means within the same column differ significantly (P≤0.05)

^1^Turkeys in treatment 10 MnO received 10 mg/kg MnO, 50 MnO received 50 mg/kg MnO, 100 MnO received 100 mg/kg MnO, 10 NP-Mn_2_O_3_ received 10 mg/kg Mn_2_O_3_ nanoparticles, 50 NP-Mn_2_O_3_ received 50 mg/kg Mn_2_O_3_ nanoparticles, 100 NP-Mn_2_O_3_ received 100 mg/kg Mn_2_O_3_ nanoparticles.

ALT—alanine aminotransferase, ALP—alkaline phosphatase, GGT—gamma-glutamyl transferase, UA- uric acid, UREA—urea, TC—total cholesterol, TG—triacylglycerols, GLU—glucose, TP—total protein, CREAT—creatinine, Ca—calcium, P—phosphorus

* SEM—for interaction D x S

**Table 4:** Indicators of redox status in the blood of turkeys receiving different sources of manganese.

Treatment^1^	Cp U/L	Mn-SOD U/gHb	GPx U/g Hb	CAT U/gHb	TAS μmol/L	GSH+GSSG μmol/L	VIT C μmol/L	ALB mg/L	LOOH μmol/L	MDA μmol/L
10 MnO	2.220^b^	510.9	85.45	428.1	443.6	0.092^b^	72.64	7.8	63.86^a^^b^	0.894^b^
50 MnO	1.448^b^^c^	697.9	91.24	472.4	429.2	0.134^a^^b^	67.78	8.0	52.90^b^^c^	1.313^a^
100 MnO	1.005^c^	774.2	100.39	498.5	391.5	0.085^b^	64.47	9.1	69.01^a^	0.966^b^
10 NP-Mn_2_O_3_	1.815^b^	419.7	87.67	364.0	434.3	0.163^a^	65.14	9.3	47.32^c^	0.846^b^
50 NP-Mn_2_O_3_	3.300^a^	698.3	84.36	400.5	384.5	0.115^a^^b^	62.83	8.3	54.67^b^^c^	1.088^a^^b^
100 NP-Mn_2_O_3_	1.125^c^	763.8	106.33	481.2	371.2	0.150^a^	68.80	9.7	73.28^a^	1.284^a^
SEM*	0.240	28.67	3.27	24.13	26.19	0.016	5.587	0.515	3.587	0.091
Dosage										
10	2.018^a^	465.3^c^	86.56^b^	396.0^b^	438.9	0.127	68.89	8.6	55.59^b^	0.870^b^
50	2.374^a^	698.1^b^	87.80^b^	436.5^a^^b^	406.8	0.125	65.31	8.2	53.79^b^	1.200^a^
100	1.065^b^	769.0^a^	103.36^a^	489.9^a^	381.3	0.118	66.64	9.4	71.15^a^	1.125^a^
SEM	0.169	20.27	2.31	17.06	18.52	0.011	3.951	0.394	2.537	0.064
Source										
MnO	1.558^b^	661.0	92.36	466.3^a^	421.4	0.104^b^	68.30	8.3	61.93	1.058
NP-Mn_2_O_3_	2.080^a^	627.3	92.79	415.2^b^	396.7	0.142^a^	65.59	9.1	58.42	1.073
SEM	0.138	16.55	1.89	13.93	15.12	0.009	3.226	0.297	2.071	0.052
*P*-value										
Dosage (D)	<0.001	<0.001	<0.001	0.005	0.156	0.848	0.845	0.106	<0.001	0.006
Source (S)	0.021	0.205	0.888	0.025	0.306	0.010	0.598	0.091	0.290	0.857
Interaction D x S	<0.001	0.305	0.209	0.554	0.826	0.023	0.612	0.508	0.025	0.032

^a, b, c^ means within the same column differ significantly (P≤0.05)

^1^Turkeys in treatment 10 MnO received 10 mg/kg MnO, 50 MnO received 50 mg/kg MnO, 100 MnO received 100 mg/kg MnO, 10 NP-Mn_2_O_3_ received 10 mg/kg Mn_2_O_3_ nanoparticles, 50 NP-Mn_2_O_3_ received 50 mg/kg Mn_2_O_3_ nanoparticles, 100 NP-Mn_2_O_3_ received 100 mg/kg Mn_2_O_3_ nanoparticles.

Mn-SOD—superoxide dismutase, GPx—glutathione peroxidise, CAT—catalase, Cp—ceruloplasmin, GSH+GSSG—total glutathione, TAS—total antioxidant status, ALB—albumin, VIT C—vitamin C, albumin—ALB, LOOH—lipid hydroperoxide, MDA—malondialdehyde

* SEM—for interaction D x S

**Table 5:** Indicators of immune status and apoptosis in the blood of turkeys receiving different sources of manganese.

Treatment^1^	IgA ng/ml	IgY ng/ml	IgM ng/ml	IL-6 pg/ml	Casp 3 pg/ml	Casp 8 ng/ml
10 MnO	22.21^b^	1074.7	474.7^c^	4.402	30.96	4.736^c^
50 MnO	18.35^b^	1121.1	551.0^b^	4.793	50.17	6.065^a^^b^
100 MnO	30.73^a^	1131.3	461.2^c^	4.809	43.04	6.399^a^
10 NP-Mn_2_O_3_	22.48^b^	1056.7	642.5^a^	4.607	43.67	4.658^c^
50 NP-Mn_2_O_3_	22.37^b^	1140.1	446.2^c^	4.657	59.15	4.870^c^
100 NP-Mn_2_O_3_	22.63^b^	1008.5	548.9^b^	5.062	46.28	5.732^b^
SEM*	2.080	28.638	22.722	0.203	1.748	0.197
Dosage						
10	22.35^a^^b^	1065.7	558.6^a^	4.505	37.32^c^	4.697^c^
50	20.36^b^	1130.6	498.6^b^	4.725	54.66^a^	5.467^b^
100	26.68^a^	1069.9	505.1^b^	4.935	44.66^b^	6.066^a^
SEM	1.471	20.250	16.102	0.143	1.236	0.139
Source						
MnO	23.77	1109.0	495.6^b^	4.668	41.39^b^	5.733^a^
NP-Mn_2_O_3_	22.49	1068.4	545.9^a^	4.775	49.70^a^	5.086^b^
SEM	1.201	16.534	13.148	0.117	1.009	0.114
P-value						
Dosage (D)	0.029	0.088	0.045	0.176	<0.001	<0.001
Source (S)	0.507	0.128	0.020	0.564	<0.001	0.001
Interaction D x S	0.037	0.083	<0.001	0.647	0.062	0.050

^a, b, c^ means within the same column differ significantly (P≤0.05)

^1^Turkeys in treatment 10 MnO received 10 mg/kg MnO, 50 MnO received 50 mg/kg MnO, 100 MnO received 100 mg/kg MnO, 10 NP-Mn_2_O_3_ received 10 mg/kg Mn_2_O_3_ nanoparticles, 50 NP-Mn_2_O_3_ received 50 mg/kg Mn_2_O_3_ nanoparticles, 100 NP-Mn_2_O_3_ received 100 mg/kg Mn_2_O_3_ nanoparticles.

IgA—immunoglobulin A, IgY—immunoglobulin Y, IgM—immunoglobulin M, IL-6—interleukin 6 Casp 3- caspase 3; Casp 8- caspase 8

* SEM—for interaction D x S

### Effect of manganese dosage

The results of two-way ANOVA showed that reducing Mn supplementation in the diet of turkeys from 100 to 50 mg/kg of feed, and then to 10 mg/kg of feed, irrespective of the form of Mn used, increased the plasma content of Mn (*P*<0.001) and decreased the following: ALP activity (*P*<0.001), content of CREAT (*P* = 0.018) and P (*P*<0.001) (Table 3), activity of Mn-SOD and GPx (*P*<0.001) and CAT (*P* = 0.005) (Table 4), and the level of Casp 8 (*P*<0.001) (Table 5).

The results of two-way ANOVA showed interactions between dosage and form in the case of Cp activity (*P*<0.001) and the content of GSH+GSSG (*P* = 0.023), LOOH (*P* = 0.025), MDA (*P* = 0.032) (Table 4), IgA (*P* = 0.037) and IgM (*P*<0.001) (Table 6) in the plasma and for CAT activity (*P*<0.001) and the content of GSH+GSSG (*P* = 0.047), LOOH (*P* = 0.016) and MDA (*P* = 0.024) in the liver (Table 6). The results of one-way ANOVA showed that reducing Mn in the turkey diet from 100 to 50 mg/kg, in the form of both MnO and NP-Mn_2_O_3_, resulted in a decrease in plasma LOOH content, whereas decreasing Mn supplementation to 10 mg/kg reduced LOOH content only when the NP-Mn_2_O_3_ form was used. A reduction in Mn in the form of MnO from 100 to 50 mg/kg in the diet resulted in an increase in plasma MDA content, while in the case of NP-Mn_2_O_3_, a reduction from 100 to 50 and then to 10 mg/kg resulted in a decrease in MDA content (Table 4).

**Table 6:** Content of redox status indicators in the liver of turkeys receiving different sources of manganese.

Treatment^1^	Mn-SOD U/g protein	CAT U/g protein	GSH+GSSG μmol/kg	VIT C μmol/kg	LOOH μmol/ kg	MDA μmol/kg
10 MnO	5.26	103.37^a^	0.897^a^	90.30	1.289^b^^c^^d^	7.64^a^
50 MnO	5.81	98.72^a^	0.821^a^	85.33	1.690^a^	7.86^a^
100 MnO	5.60	85.18^b^	0.721^b^	85.11	1.542^a^^b^	5.64^b^
10 NP-Mn_2_O_3_	6.77	83.75^b^	0.861^a^	88.70	1.683^a^	7.18^a^
50 NP-Mn_2_O_3_	6.34	90.79^b^	0.891^a^	89.37	1.627^a^^d^	7.86^a^
100 NP-Mn_2_O_3_	5.14	90.46^b^	0.830^a^	82.04	1.211^b^^c^	7.27^a^
SEM*	0.465	2.762	0.029	3.028	0.122	0.387
Dosage						
10	6.02	93.56^a^	0.879^a^	89.50	1.486	7.41^a^
50	6.08	94.75^a^	0.856^a^	87.35	1.658	7.86^a^
100	5.37	87.82^b^	0.776^b^	83.57	1.377	6.45^b^
SEM	0.329	1.953	0.021	2.141	0.086	0.274
Source						
MnO	5.56	95.76	0.813	86.91	1.507	7.04
NP-Mn_2_O_3_	6.08	88.33	0.861	86.70	1.507	7.44
SEM	0.268	1.594	0.017	1.748	0.071	0.224
P-value						
Dosage (D)	0.255	0.034	0.002	0.150	0.077	0.002
Source (S)	0.173	0.002	0.052	0.933	1.000	0.218
Interaction D x S	0.117	<0.001	0.047	0.469	0.016	0.024

^a, b, c, d^ means within the same column differ significantly (P≤0.05)

^1^Turkeys in treatment 10 MnO received 10 mg/kg MnO, 50 MnO received 50 mg/kg MnO, 100 MnO received 100 mg/kg MnO, 10 NP-Mn_2_O_3_ received 10 mg/kg Mn_2_O_3_ nanoparticles, 50 NP-Mn_2_O_3_ received 50 mg/kg Mn_2_O_3_ nanoparticles, 100 NP-Mn_2_O_3_ received 100 mg/kg Mn_2_O_3_ nanoparticles.

Mn-SOD—superoxide dismutase, CAT—catalase, GSH+GSSG—total glutathione, VIT C—vitamin C, LOOH—lipid hydroperoxide, MDA—malondialdehyde

* SEM—for interaction D x S

The reduction in Mn supplementation in the form of MnO from 100 to 50 and then to 10 mg/kg increased the content of MDA and GSH + GSSG as well as CAT activity, while the reduction of the NP-Mn_2_O_3_ supplement, to 50 and to 10 mg/kg, had no effect on the content of MDA or GSH + GSSG or on CAT activity, but increased the level of LOOH in the liver (Table 6).

A reduction in the level of IgA in the turkey blood was induced by decreasing the addition of Mn in the form MnO from 100 to 50, and also to 10 mg/kg of diet, whereas the corresponding reduction of Mn in the form of NP-Mn_2_O_3_ did not affect the level of this immunoglobulin. The results of one-way ANOVA showed that the content of immunoglobulin M following the use of 50 mg/kg increased in the case of the MnO, but decreased for NP-Mn_2_O_3_. The reduction in Mn in the form of NP-Mn_2_O_3_ from 100 to 10 mg/kg caused an increase in the IgM level in the blood, but this effect was not observed when the same amount of Mn was administered in the form of MnO. The reduction in Mn supplementation in the form of NP-Mn_2_O_3_ from 100 to 50 and to 10 mg/kg resulted in an increase in Cp activity in the blood, while the reduction in Mn in the form of MnO caused an increase in the activity of this enzyme only in the case of 10 mg/kg diet (Table 5).

## Discussion

Manganese is an element essential to the proper functioning of the antioxidant and immune system [36]. For this reason poultry diets must be supplemented with this element, but in a properly selected form and quantity that does not cause adverse health effects. Our study showed that reducing Mn supplementation from 100 to 50 or 10 mg/kg of feed, irrespective of the form used, resulted in greater content of this element in the plasma of the turkeys. The pool of absorbed manganese is transported into the liver cells, where it is located in the mitochondrion, the cell nucleus, incorporated into the newly synthesized proteins and occurs as the free Mn^2+^. Excess manganese is absorbed by cell lysosomes, with which it is then transferred to the bile duct and excreted in the bile [37]. With long-term administration of higher doses of Mn, the body gets used to the constant supply of this element, therefore Mn does not accumulate. It is likely that the Mn deficiency in the diet led to its increased absorption in the body as an adaptive mechanism to the low intake of this element. Lebda et al. [38] observed an increase in plasma Mn content in rats, but the diet of these animals was additionally enriched with 6 mg Mn/kg BW/day.

Our research shows that both the reduction of Mn addition and the substitution of MnO with Mn nanoparticles did not affect most of the biochemical blood indices. The reduction of the Mn addition to the turkey diet, however, resulted in a decrease in the CREAT and P content in the blood plasma. According to a study on Japanese quail, increasing Mn to diet results in increased CREAT content in the blood [39]. Mn is necessary for the proper metabolism of creatinine with the enzyme creatininase [40]. From research carried out by Bozkurt et al. (2015) on broiler chickens treated with MnO and Mn chelates with methionine, it appears that both the Mn form used and the dose did not affect the P level in chickens’ blood.

Research carried out by Eidi et al. [41] indicates that an excess of Mn in the diet may adversely affect liver function, as evidenced by increased ALP activity in the plasma of rats. Our study showed that a decrease in Mn supplementation, irrespective of the form used, decreased ALP activity, whereas replacing MnO with NP-Mn_2_O_3_ resulted in increased GGT activity. A reduction in liver enzymes was also observed by Yuan et al. [42] in the blood of chickens in which Mn in the diet was reduced.

In the present study, the reduction in Mn supplementation in the form of NP-Mn_2_O_3_ from 100 to 50, and even to 10 mg/kg of turkey diet was found to decrease the intensity of lipid peroxidation reactions, which was manifested as a decrease in LOOH and MDA in the plasma. Although elevated levels of LOOH were observed in the liver when NP-Mn_2_O_3_ was reduced from 100 to 50 and 10 mg/kg of diet, no increase in MDA content was noted. The changes observed in parameters illustrating the intensity of oxidation processes indicate that the antioxidant system functioned properly when the Mn additive in the form of nanoparticles was reduced, which is also indicated by the decrease in the activity of Mn-SOD, GPx and CAT. Superoxide dismutase (SOD) catalyses the superoxide anion radical dismutation reaction to H_2_O_2_, which is then broken down by glutathione peroxidase (GPx) into water. At high concentrations of H_2_O_2_, it is additionally degraded to water and molecular oxygen by catalase (CAT) [19]. When the NP-Mn_2_O_3_ supplement was reduced, lipid oxidation processes may have increased slightly, resulting in the mobilization of antioxidant enzymes and the use of reserves of enzyme cofactors (e.g. trace elements Mn, Zn, Cu and Fe), and in consequence a reduction in their activity. A reduction in the activity of antioxidant enzymes can also be observed in the case of oxidative stress induced by excess Mn in cells. Research carried out by Erikson et al. [36] and Liu et al. [19] indicates that the use of higher levels of Mn than recommended resulted in a decrease in the activity of antioxidant enzymes Mn-SOD and GPx, accompanied by an increase in the plasma content of MDA in chickens. The authors concluded that the changes in redox status parameters indicate an increase in oxidative stress. Lu et al. [43] and Li et al. [25] found that the addition of higher than recommended levels of Mn to the diet of chickens increases expression of the Mn-SOD gene, and thus increases Mn-SOD activity in various tissues. Bai et al. [44] also found that increased addition of Mn to the diet of laying hens increases Mn-SOD activity. In a study on chickens, Bozkurt et al. [3] found that increasing Mn supplementation in the diet to above the recommended level stimulates the antioxidant system and reduces lipid peroxidation mechanisms. Fouad et al. [45] found no effect of increasing Mn supplementation to 90 mg/kg diet on the plasma MDA level or SOD activity in ducks.

Our study, in which the addition of MnO to the turkey diet was reduced to 50 and then to 10 mg/kg, showed increased MDA content accompanied by reduced LOOH content and decreased Mn-SOD, GPx and CAT activity in the blood. Similarly, Luo et al. [46] observed that reducing the addition of Mn to the diet of growing chickens resulted in a decrease in Mn content in tissues and in Mn-SOD activity in the heart. Our research indicates that reducing the addition of MnO to the turkey diet intensified lipid peroxidation processes more than in the case of NP-Mn_2_O_3_, as evidenced by the increased MDA content in the plasma and liver and increased CAT activity in the liver.

Although Mn plays a key antioxidant function in the body, a high Mn level may induce oxidative stress, which may exacerbate apoptosis [47]. Excess Mn can accumulate in the cellular mitochondrion, thereby increasing the level of oxidants and the release of cytochrome c. Released cytochrome c leads to the activation of Casp 8 and 9, which activate apoptosis executor protein Casp 3. Mn accumulated in the endoplasmic reticulum increases the level of proteins such as Bip, PERK, Bima and Bax, which activate Casp 12, which in turn also activates Casp 3. There are also cellular mechanisms by which this element directly activates the caspase cascade. Mn accumulated in the cytoplasm of the cell activates PKCδ kinase, which activates Casp 8, which in turn activates Casp 3 [36, 48]. Our research showed that replacing Mn in the form of MnO by NP-Mn_2_O_3_, irrespective of the amount added, resulted in a decrease in the level of total Casp 8 and an increase in total Casp 3. Although our research did not indicate that replacing MnO in the turkey diet with Mn in the form of NP-Mn_2_O_3_ induces lipid oxidation, this does not mean that it does not increase oxidation, e.g. of proteins, DNA or sugars. Reactive oxygen species generated by the oxidation of these molecules may also induce oxidative stress in the cell, followed by a cascade of caspases initiating (Casp 9 and 12) and executing (Casp 3) apoptosis. Our study also showed that, irrespective of the form of Mn, a reduction from 100 to 50 and to 10 mg/kg of turkey diet resulted in a decrease in the plasma level of Casp 8. In addition, reducing the addition of both forms of Mn to 10 mg/kg resulted in a decrease in the level of Casp 3. Caspase 3 activity is unique to apoptosis, as it does not occur in other forms of cell death and provides strong evidence for the presence of apoptosis [49]. The total caspase 3 and 8 increase doesn’t correlate to apoptosis increase, and only cleaved ones are activated, related to apoptosis, and used as apoptosis markers. According to Smith et al. [48] Mn induces caspase-dependent apoptosis. Caspases are endoproteases that initiate (8 and 9) and execute (3, 6 and 7) apoptotic events. Cell death pathways through caspase-3 cleavage seem to be the predominant mode of Mn-dependent caspase-induced apoptosis in neuronal cells. In addition to caspase-3 cleavage, Mn also increases caspase 3-promoter activity through the Sp1 binding regions in PC-12 cells treated with 0.25 mM–1mM MnCl2 for 18 h, thereby increasing caspase-3 mRNA and proteins. Our studies did not investigate the level of cleaved caspase, but the increase in the total caspase 3 (apoptosis executive protein) due to the use of Mn nanoparticles allows us to assume the activation of cellular apoptosis. Manganese also plays a key role in the removal of reactive oxygen species produced by immunologically active cells, e.g. macrophages in the process of cell phagocytosis. The available literature offers little information on the effect of manganese deficiency on the development and functioning of the immune system [50]. Research carried out by Liu et al. [16] indicates that excessive exposure to Mn can cause it to accumulate in immunocompetent organs. Excess manganese can disturb the balance of trace elements in the lymphatic organs and induce immune suppression at the molecular level. In our study, reducing the addition of MnO to the turkey diet resulted in a decrease in plasma IgA levels. On the other hand, the decrease in Mn supplementation in the form of both MnO and NP-Mn_2_O_3_ increased plasma IgM levels, especially when Mn was reduced to 10 mg/kg of diet. An increased level of IgM is most often associated with the primary immune response to exposure to an immunogen or pathogen [51]. Induction of IgA may occur against a background of constant antigenic challenge from food, environmental antigens and numerous commensal microorganisms [52]. Specific IgA antibodies have been shown to provide effective protection against a range of invading pathogens, including viruses, bacteria and protozoa, and their products, such as toxins [53].

There are reports indicating that the addition of Mn to the diet in amounts greater than the animals’ requirement for this element increases phagocytosis of macrophages and NK cells, as well as expression of pro-inflammatory cytokines IL-1β, IL-6, IL-8 and IFN-γ [54, 55] and antibody titres in chickens [56]. Sunder et al. [57] found that Mn supplementation at a level of 100 mg/kg diet increased immunity in chickens. Gajula et al. [56] also noted that an increased Mn level in the diet of chickens stimulated the immune response, and Oweson et al. [58] showed that accumulation of Mn in animal tissues improved immune defence. Our research indicates that neither the replacement of the MnO supplement in the turkey diet with Mn in the form of NP-Mn_2_O_3_ nor the reduction in the amount of either form added to the diet affected the level of IL-6 in the blood. The level of interleukin IL-6, sometimes referred to as B-cell growth factor, which has a regenerative or anti-inflammatory effect, increases mainly during inflammatory states in the body [59].

An increased level of ceruloplasmin as an acute phase protein also may be a cellular response to oxidative stress, and consequently to inflammation [60]. In our study, replacing Mn in the form of MnO with NP-Mn_2_O_3_ in the turkey diet had no effect on Cp activity, but a reduction in the amount of both forms of added Mn, especially to 10 mg/kg of diet, resulted in increased activity of this protein. What is more, the increase in Cp activity was associated with an increase in the plasma Mn content in the groups with reduced supplementation of this element. Research carried out by Khandelwal et al. [61] suggests a high interaction between Mn and Cu. The authors found that high Mn exposure inhibits Cu absorption and affects the content of immunoglobulins. Khandelwal et al. [61] also showed that a sufficiently high content of Cu in the body helps to reduce accumulation of Mn in order to reduce the toxicity of this element. A reduction in Mn exposure may therefore increase the absorption of Cu in the body. This dependence may explain the increased Cp activity noted in our research, which depended on the Cu content. Its activity is increased by a high level of this element in the body [60]. In the context of the lack of increased plasma IL-6 content in the turkeys in our research, with increased levels of Cp and IgM, it is difficult to draw definitive conclusions on the negative effect of either the replacement of MnO by NP-Mn_2_O_3_ or the reduction of this additive in the diet (especially to 50 mg/kg) on the immune system of turkeys. Since the changes in IgM concentration were not correlated with an increase in IL-6 concentration, the increase in IgM was probably not due to stimulation of phagocytes.

The present study found that replacing MnO with NP-Mn_2_O_3_ had no effect on the growth performance of young turkeys, but contributed to lower feed consumption per kg body weight. A study by Berta et al. [62], in which different levels of manganese were added to chicken feed in the form of MnO and Mn-fumarate, also did not confirm an effect of these additives on growth performance in chickens.

## Conclusions

The study showed that irrespective of the form of Mn used, reducing the Mn level recommended by British United Turkeys for supplementation of the diet of young turkeys from 100 mg/kg to 10 mg/kg increases the content of this element in the blood with no adverse effect on growth performance or the immune system.

The reduction in Mn supplementation in the form of NP-Mn_2_O_3_ from 100 to 50 and even to 10 mg/kg of turkey diet has no negative effect on antioxidant defence in young turkeys. A 50% reduction of the recommended Mn level in the form of MnO enhances lipid oxidation processes.

Replacing MnO with NP-Mn_2_O_3_ in the turkey diet probably can increase apoptosis in young turkeys. On the other hand, irrespective of the form of Mn used, reducing supplementation of the turkey diet with this element from 100 to 50 and even to 10 mg/kg probably can reduce apoptosis.
